# P-470. Epidemiology of Invasive Fungal Disease in Pediatric Acute Lymphoblastic Leukemia

**DOI:** 10.1093/ofid/ofaf695.685

**Published:** 2026-01-11

**Authors:** Caroline Maguire, Caitlin N Brammer, Justin Markham, Hilary Miller-Handley, Mark Murphy, Grant C Paulsen, Lara A Danziger-Isakov, William R Otto

**Affiliations:** Cincinnati Children's Hospital Medical Center, Cincinnati, OH; Cincinnati Children's Hospital Medical Center, Cincinnati, OH; Cincinnati Children's Hospital Medical Center, Cincinnati, OH; Cincinnati Children's Hospital Medical Center, Cincinnati, OH; Cincinnati Children's Hospital Medical Center, Cincinnati, OH; Cincinnati Children's Hospital Medical Center, Cincinnati, OH; Cincinnati Children's Hospital, Cincinnati, OH; Cincinnati Children's Hospital Medical Center, Cincinnati, OH

## Abstract

**Background:**

Invasive fungal disease (IFD) is an important cause of morbidity and mortality in pediatric acute lymphoblastic leukemia (ALL) patients. Risk of IFD is variable in children with ALL, and need for antifungal prophylaxis varies. There are few epidemiologic studies of IFD in ALL patients. This study sought to define the epidemiology of IFD and evaluate use of antifungal prophylaxis in ALL.

**Table 1:**
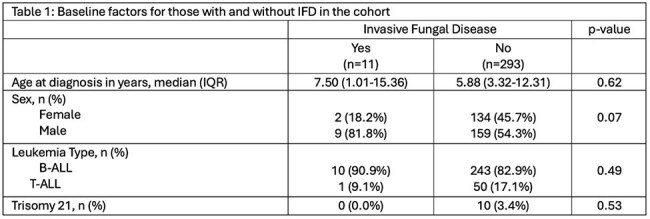
Baseline demographic and clinical characteristics for those with and without IFD in the cohort

**Table 2:**
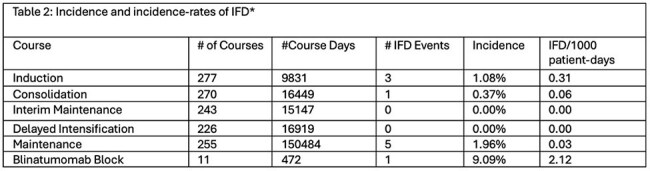
Incidence and incidence-rates of ifD

**Methods:**

This was a retrospective study of patients with de novo ALL treated at Cincinnati Children’s Hospital Medical Center from 1/1/2012-12/31/2022. Clinical and microbiology data was abstracted from the medical record. Proven and probable IFD was identified using MSG/EORTC definitions. The incidence and incidence-rates of IFD were calculated. Days of antifungal therapy were collected for each chemotherapy course.

**Table 3:**
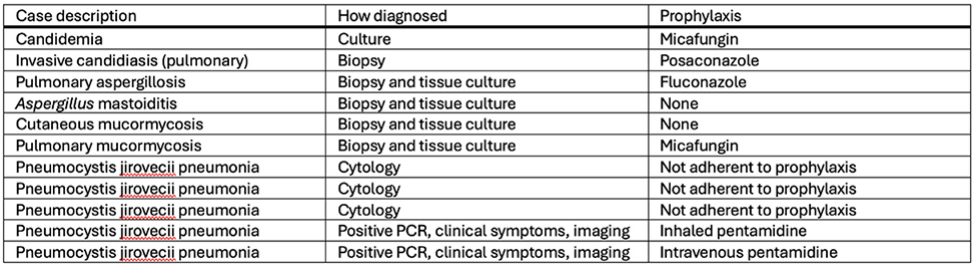
Cases of proven or probable IFD in the cohort

**Figure 1:**
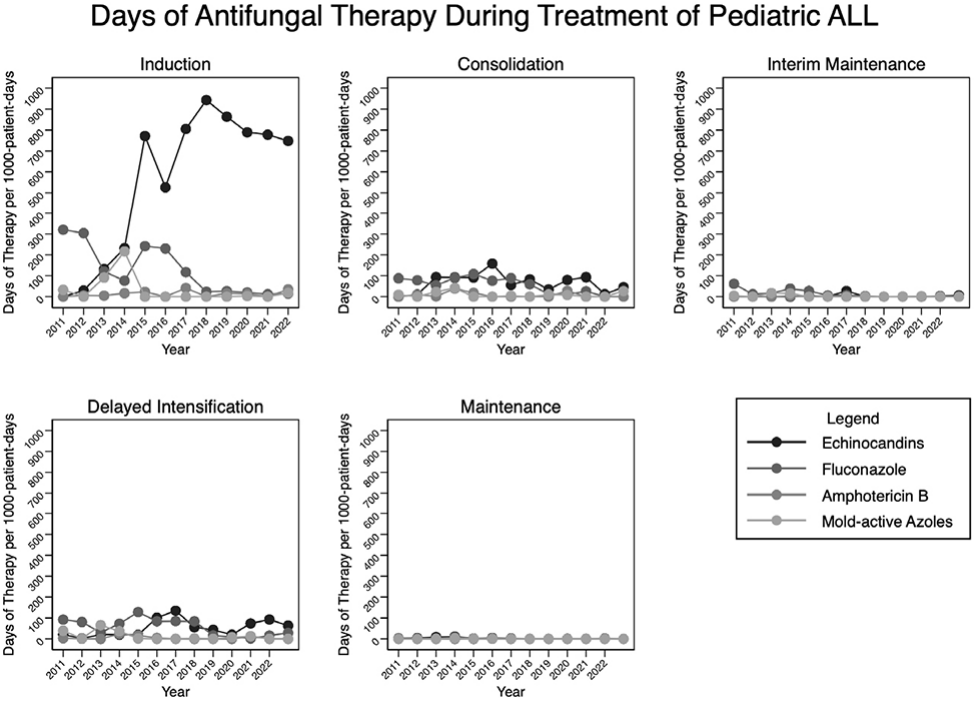
Days of antifungal therapy during treatment of pediatric ALL

**Results:**

A total of 11/304 (3.6%) patients had proven (n=9)/probable (n=2) IFD during ALL treatment. There were no differences in age, sex, or leukemia type for those with and without IFD (Table 1). Invasive mold infections accounted for 4/11 cases of IFD, with 3 cases occurring during Induction and 1 case during Consolidation (Table 2). Cases are shown in Table 3. Two infections with yeasts occurred, biopsy-proven invasive candidiasis during blinatumomab therapy and candidemia during an OCTADAD course of Interfant-06. Five cases of proven/probable *Pneumocystis jiroveci* pneumonia occurred during maintenance chemotherapy. No IFD cases occurred in interim maintenance or delayed intensification. For routine ALL chemotherapy courses, the IFD incidence-rate was highest during Induction (Table 2). Prophylaxis was frequently administered during Induction, with lower prescribing rates during other courses (Figure 1). Over the course of the study, the predominant prophylactic agent changed from fluconazole to micafungin.

**Conclusion:**

IFD was uncommon in this single-center ALL cohort. Most episodes of IFD occurred during intensive chemotherapy courses, consistent with guidelines that children with ALL should receive prophylaxis during Induction. A majority of patients with PJP were not adherent to prophylaxis, highlighting its importance. Over the past decade, echinocandins have become to predominant antifungal prophylactic agents at our institution.

**Disclosures:**

Grant C. Paulsen, MD, Moderna, Inc: Grant/Research Support|Pfizer: Grant/Research Support|Sanofi: Grant/Research Support Lara A. Danziger-Isakov, MD, MPH, Aicuris: Grant/Research Support|Ansun BioPharma: Grant/Research Support|Astellas: Advisor/Consultant|Astellas: Grant/Research Support|Merck: Advisor/Consultant|Merck: Grant/Research Support|Pfizer (Any division): Grant/Research Support|Takeda: Grant/Research Support

